# Noise-induced transitions and shifts in a climate–vegetation feedback model

**DOI:** 10.1098/rsos.171531

**Published:** 2018-04-11

**Authors:** Dmitri V. Alexandrov, Irina A. Bashkirtseva, Lev B. Ryashko

**Affiliations:** Department of Theoretical and Mathematical Physics, Laboratory of Multi-Scale Mathematical Modeling, Ural Federal University, Ekaterinburg 620000, Russian Federation

**Keywords:** nonlinear dynamics, noise, stochastic fluctuations

## Abstract

Motivated by the extremely important role of the Earth’s vegetation dynamics in climate changes, we study the stochastic variability of a simple climate–vegetation system. In the case of deterministic dynamics, the system has one stable equilibrium and limit cycle or two stable equilibria corresponding to two opposite (cold and warm) climate–vegetation states. These states are divided by a separatrix going across a point of unstable equilibrium. Some possible stochastic scenarios caused by different externally induced natural and anthropogenic processes inherit properties of deterministic behaviour and drastically change the system dynamics. We demonstrate that the system transitions across its separatrix occur with increasing noise intensity. The climate–vegetation system therewith fluctuates, transits and localizes in the vicinity of its attractor. We show that this phenomenon occurs within some critical range of noise intensities. A noise-induced shift into the range of smaller global average temperatures corresponding to substantial oscillations of the Earth’s vegetation cover is revealed. Our analysis demonstrates that the climate–vegetation interactions essentially contribute to climate dynamics and should be taken into account in more precise and complex models of climate variability.

## 1. Introduction

Whether or not all physical mechanisms and their correlations were determined or described in detail, the fact that they relate the climate and land surface changes was observed and reported even many years ago. So, for instance, the notes written by Thomas Jefferson that the Virginia winters were getting noticeably warmer [1] shortly thereafter were described as a result of land clearing and cultivation [2]. Alexander von Humbolt apparently was one of the first scientists paying attention to investigation of the interplay between the climate and vegetation changes [3].

It is now conventional wisdom that the Earth’s climate determines the world’s vegetation, which has often been considered as a climate visualization [4]. However, the opposite influence of vegetation on the planetary climate is of primary importance too [5–8]. Here, among others, deforestation experiments for the Amazon [9–12], desertification experiments for the Sahel [13,14] and experiments for the boreal forest [15,16] may be mentioned.

An important point is that the effect of deforestation leads to different aspects of climate change such as biogeophysical effects of surface albedo and roughness changes, changes in the energy fluxes and cloud cover altering the atmospheric temperature. Generally speaking, the ice–albedo feedback represents the climate phenomenon of surface albedo changes caused by changes in the sea ice and snow-covered areas. Such changes in albedo amplify the initial changes in the ice and snow areas. So, for example, cooling increases the ice cover and hence the albedo. This process in turn decreases the amount of solar energy absorbed and results in more cooling. In the case of boreal forest, the climate warming is connected with the surface albedo decreasing as a result of snow masking in forests and sea ice melting due to the heat flux from vegetated land to polar seas. In addition, tropical deforestation and changes in the vegetation cover have a direct impact on global atmospheric circulations [17,18]. Note that deforestation can be amplified by different anthropogenic changes in the land cover as well as forest fires and pest activities.

An important role in climate change is connected with the biogeochemical effect reflecting the interactions between the biosphere and the chemical composition in the Earth’s atmosphere. So, for example, increasing the atmospheric CO_2_ concentration builds up the Earth’s biomass as plant productivity becomes higher, until a saturation state is attained [19]. Human activity also brings a great contribution to the biogeochemical cycle. It is responsible for the increased level of atmospheric greenhouse gases which, in turn, absorb the long-wave radiation outgoing from the Earth’s surface and, as a result, the lower atmosphere becomes warmer. The atmospheric carbon dioxide in this respect apparently has the greatest influence on the Earth’s climate [20]. Its concentration has increased from approximately 260–270 ppm in the pre-industrial era [21] to a level of 378 ppm in 2005 (https://climate.nasa.gov/vital-signs/carbon-dioxide/) and then to its current value of approximately 407 ppm in 2018 (https://climate.nasa.gov/vital-signs/carbon-dioxide/).

What is more, this present day CO_2_ concentration more than twice exceeds the local maxima of CO_2_ repetitive behaviour over the last 400 000 years (https://climate.nasa.gov/vital-signs/carbon-dioxide/). An important point is that there is a strong correlation between the carbon dioxide concentration in the atmosphere and the Earth’s climate [22].

Thus, the land–atmosphere interactions link the carbon dioxide cycle (CO_2_ assimilation and diffusion into the leaves and water vapour through transpiration) and water use in plants. This mechanism is very sensitive to light intensity, air temperature, precipitation, soil–water content, plant diversity and atmospheric carbon dioxide concentration which would lead to an unstable climate dynamics [7,23–26]. For example, this occurs when the climate system undergoes an abrupt transition from a disappearing green state to a desert state [23]. In other words, the climate–vegetation system may have different equilibria (attractors) and migrate between them under the influence of different external factors such as natural and anthropogenic forcing [27,28]. Therefore, a necessary step is to study the sensitivity of the climate–vegetation system to past-time variability induced by external forcing.

Taking all the aforesaid into consideration let us underline the dominant role of climate changes on terrestrial vegetation (e.g. evolution of grassland and forests), on the biogeochemical cycle including the carbon dioxide interaction with biomass and soil as well as on the amount of some species and potential disappearance of others [29–33]. The vegetation feedback involves in particular the alteration of surface albedo, land roughness and atmospheric composition of greenhouse gases. These mechanisms are responsible for the evolutionary behaviour of the very sensitive and complex climate–vegetation system. This paper is devoted to the influence of external stochastic forcing in a simple climate–vegetation model leading to drastically new dynamic phenomena and demonstrating possible abrupt changes in its evolutionary behaviour.

## 2. Climate–vegetation feedback model

To determine the role of stochastic forcing on the climate–vegetation interaction, let us use the simple conceptual mathematical model developed by Rombouts & Ghil [34]. This model contains the governing equations for the global average temperature *T* and the fraction of land *A* covered by vegetation. The temperature *T* changes with time *t* as a result of balance between the incoming and outgoing energy fluxes:

2.1 $$C_{T}\frac{dT}{dt}=(1-\alpha (T,A))Q_{0}-R_{0}(T),$$

where *C*_*T*_ is the heat capacity, *Q*_0_ is the incoming solar energy and functions *α*(*T*,*A*) and *R*_0_(*T*) determine dependencies of the Earth’s albedo and the outgoing energy flux, respectively. Introducing the planet fraction *p* that is land (1−*p* is the fraction of ocean), one can express the first of these functions as

2.2 $$\alpha (T,A)=(1-p)\alpha_{o}(T)+p(\alpha_{v}A+\alpha_{g}(1-A)),$$

where *α*_*v*_ and *α*_*g*_ represent the albedo of vegetation and ground so that *α*_*v*_<*α*_*g*_ as forests and savannas absorb more energy than bare ground.

The ocean albedo *α*_*o*_ should depend on temperature *T* and, in particular, on temperatures *T*_*α*,ℓ_ and *T*_*α*,*u*_ below and above which the ocean is ice-covered and ice-free, respectively. This dependence can be represented as a ramp function of the form [35,36]

2.3 $$\alpha_{o}(T)=\left\{ \begin{array}{ll} \alpha_{\max}, & T\leq T_{\alpha ,\ell }\\ \alpha_{\max}+f(T), & T_{\alpha ,\ell }<T<T_{\alpha ,u}\\ \alpha_{\min}, & T>T_{\alpha ,u}, \end{array}\right.$$

where

$$f(T)=\frac{\alpha_{\min}-\alpha_{\max}}{T_{\alpha ,u}-T_{\alpha ,\ell }}(T-T_{\alpha ,\ell })$$

and *α*_*max*_ and *α*_*min*_ are the albedos of ice-covered and ice-free ocean.

The energy flux outgoing from the Earth takes into account the fact that increasing carbon dioxide decreases the outgoing radiation

2.4 $$R_{0}(T)=B_{0}+B_{1}(T-T_{\mathrm{opt}}),$$

where *B*_0_ and *B*_1_ are the model constants and *T*_*opt*_ is the optimal vegetation growth temperature.

The evolution of vegetation cover *A* can be described by the following logistic law:

2.5 $$\frac{dA}{dt}=\beta (T)A(1-A)-\gamma A,$$

where *γ* designates the vegetation death rate and

$$\beta (T)=\max\{0,1-k{\left(T-T_{\mathrm{opt}}\right)}^{2}\}$$

determines the parabolic growth rate in a certain temperature interval with a maximum temperature *T*=*T*_*opt*_ (here *k* represents the growth curve thickness).

The model (2.1)–(2.5) determines the evolutionary behaviour of the global average temperature and the land fraction covered by vegetation. Below we detail its deterministic dynamics and describe its new evolutionary scenario under the influence of stochastic forcing.

## 3. Deterministic dynamics

**Figure 1.**
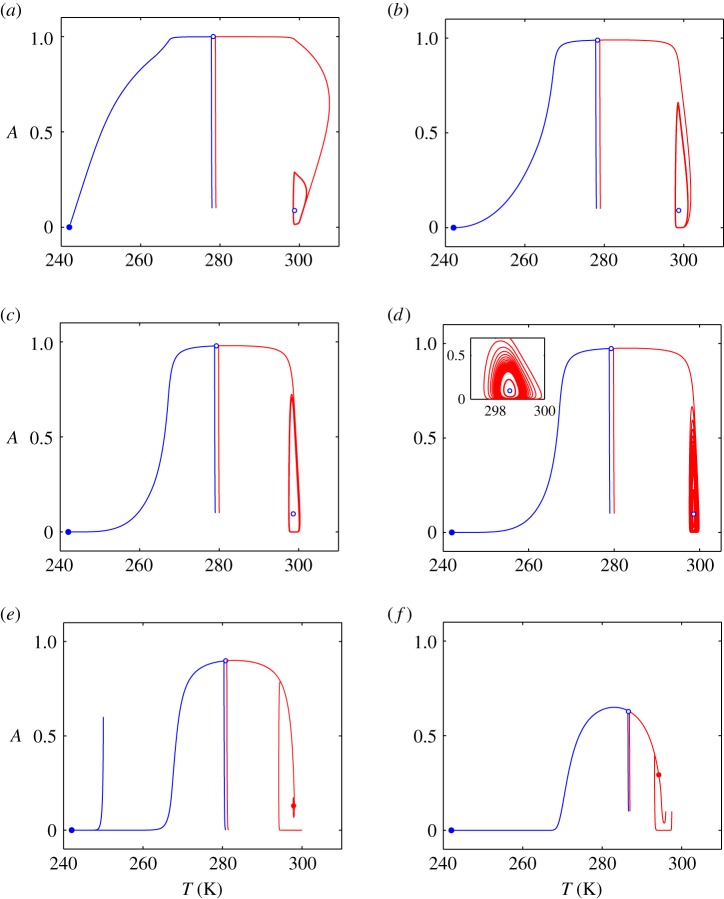
Phase trajectories of the climate–vegetation model: (*a*) *γ*=0.001, (*b*) *γ*=0.01, (*c*) *γ*=0.02, (*d*) *γ*=0.025, (*e*) *γ*=0.1, (*f*) *γ*=0.35. The model parameters are [34]: *C*_*T*_=500 *W* *yr* *K*^−1^ *m*^−2^, *Q*_0_= 342.5 *W* *m*^−2^, *p*=0.3, *α*_*v*_=0.1, *α*_*g*_=0.4, *α*_*max*_=0.85, *α*_*min*_=0.25, *T*_*α*,ℓ_=263 K, *T*_*α*,*u*_=300 *K*, *B*_0_=200 *W* *m*^−2^, *B*_1_=2.5 *W* *K*^−1^ *m*^−2^, *T*_*opt*_=283 *K*, *k*=0.004 *yr*^−1^ *K*^−2^.

A bifurcation analysis of the deterministic climate–vegetation model (2.1)–(2.5) was carried out in Rombouts & Ghil [34]. The important details of the phase portraits for this nonlinear feedback model are demonstrated in figure 1. Here, phase trajectories leading to ‘cold’ attractor (snowball) are plotted by blue colour, and others leading to ‘warm’ attractor are shown by red colour. In the first instance, the model has a stable limit cycle (depicted on the right-hand sides of figure 1*a*–*d* by the closed red lines) embracing an unstable equilibrium (shown by the open blue circles inside the limit cycles). An interesting feature of the deterministic system is its behaviour near the separatrix dividing basins of attraction of ‘warm’ and ‘cold’ attractors. As can be seen from figure 1, the trajectories starting near the separatrix move along it for some time before approaching the attractors.

Note that with further increase of the vegetation death rate *γ*, the system undergoes a supercritical Andronov-Hopf bifurcation: the limit cycle and unstable equilibrium merge, and a stable equilibrium appears (see red filled circles in figure 1*e*,*f*). In addition, there are the stable (shown on the left-hand sides of figure 1*a*–*f* by the blue filled circles) and unstable (illustrated by the open circles in the upper parts of all panels in figure 1) equilibria.

Generally speaking, there are only two opposite stable states of the climate–vegetation system under consideration: the limit cycle or stable equilibrium on the right (red colour) and the stable equilibrium on the left (blue colour). The first of them describes small climate–vegetation oscillations near the global average temperature 300 K (warm climate) whereas the second one corresponds to the snowball Earth scenario (242 K) when no fraction of land is covered by vegetation. These opposite stable regimes of climate–vegetation system are divided by the unstable equilibrium so that the phase trajectories (blue and red lines) go very close together in its vicinity (this occurs near 280 K in figure 1*a*–*e* and 286 K in figure 1*f*). In this zone, even small temperature perturbations can throw the system across its separatrix and crucially change the dynamics of the system. An important point is the essential influence of vegetation death rate on the deterministic dynamics. The possible stable climate–vegetation oscillations expressed by the stable limit cycle become narrower with increasing vegetation death rate *γ* and degenerate into just a stable point in the phase diagram.

**Figure 2.**
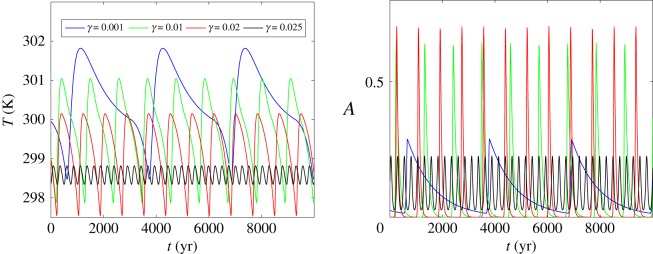
Time series of deterministic system.

The time-dependent oscillations of average temperature and vegetation fraction are shown in figure 2. As is easy to see, the period of climate–vegetation oscillations decreases with increasing vegetation death rate. The interval of temperature fluctuations therewith decreases and the interval of land fraction covered by vegetation takes a more complex behaviour in accordance with the phase portraits shown in figure 1.

## 4. Stochastic dynamics

To study the effect of external stochastic forcing on the climate–vegetation dynamics, let us consider the following nonlinear system:

4.1 $$\begin{array}{rl} C_{T}\frac{dT}{dt} & =(1-\alpha (T,A))Q_{0}-R_{0}(T)\\ \;\text{and}\;\frac{dA}{dt} & =(\beta (T)+\varepsilon \xi (t))A(1-A)-\gamma A, \end{array}\}$$

where *ξ*(*t*) is a standard Gaussian white noise with parameters 〈*ξ*(*t*)〉=0, 〈*ξ*(*t*)*ξ*(*τ*)〉=*δ*(*t*−*τ*), and *ε* designates the noise intensity (hereafter its dimension is omitted for the sake of simplicity). The stochastic system (4.1) follows from equations (2.1) and (2.5) after the replacement of deterministic growth rate *β*(*T*) by its corresponding stochastic function *β*(*T*)+*εξ*(*t*). This means that we study how diverse fluctuations induced by different natural and anthropogenic processes are able to change the vegetation growth rate and, thus, the evolutionary scenario of the climate–vegetation system overall.

**Figure 3.**
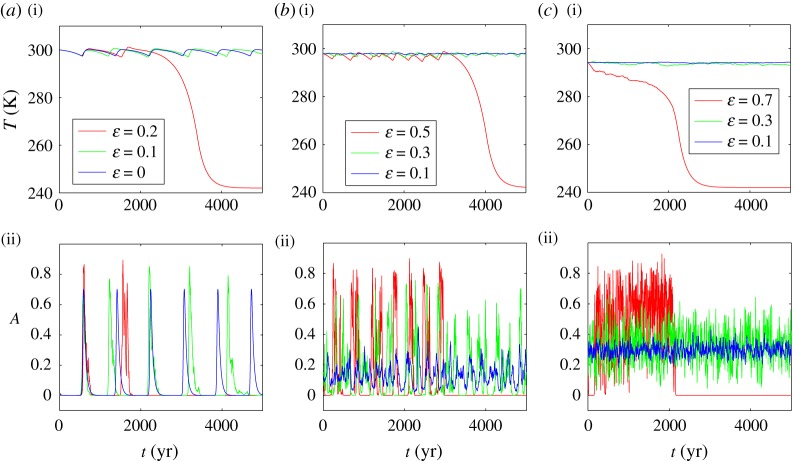
Noise-induced dynamics of the climate–vegetation model for (*a*) *γ*=0.02, (*b*) *γ*=0.1 and (*c*) *γ*=0.35.

Figure 3 shows that the additional stochastic disturbances lead to drastic changes in the system dynamics. Note that for numerical simulations of random trajectories, we have used the Euler–Maruyama scheme with time step 0.01 year. While the noise intensity *ε* is small enough (blue and green colour), in the time interval 0≤*t*≤5000 *yr*, the global temperature *T* slightly fluctuates near its mean value that is close to 300 K. In figure 3*a*, for *γ*=0.02, one can see the noisy oscillations (green) near the deterministic small-amplitude periodic trajectory (blue). In figure 3*b*,*c*, for *γ*=0.1 and *γ*=0.035, there are small-amplitude stochastic oscillations around the deterministic stable equilibria. In the presence of such small noises, the vegetation land cover *A* fluctuates near either the deterministic periodic trajectory (figure 3*a*) or the stable equilibria (figure 3*b*,*c*). Note that the amplitude of these stochastic oscillations grows with increasing noise intensity *ε*.

When the noise intensity becomes large enough, the global temperature demonstrates a sharp stepwise decrease to the Earth’s snowball state (242 K). What is more, this stepwise transition becomes steeper with increasing vegetation death rate (compare the red curves in figure 3). The level of vegetation cover *A* in the course of this transition tends to zero, as is demonstrated in figure 3*a*(ii)–*c*(ii).

**Figure 4.**
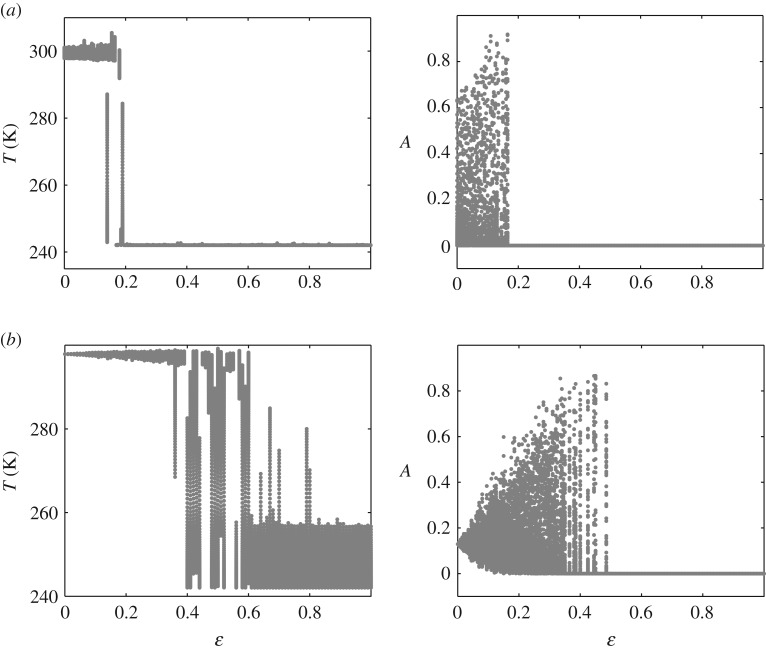
Noise-induced random states of the climate–vegetation system for (*a*) *γ*=0.01 and (*b*) *γ*=0.1.

Strictly speaking, there is a range of noise intensity leading the climate–vegetation system to its breakdown state of the snowball Earth. This range essentially depends on the vegetation death rate *γ*. It is illustrated in figure 4, where we demonstrate different random states of the system under consideration. So, for instance, the transitions occur in the interval 0.15≲ε≲0.2 for *γ*=0.01 (figure 4*a*), and 0.35≲ε≲0.55 for *γ*=0.1 (figure 4*b*). If the noise intensity exceeds this interval, the climate–vegetation system inevitably tends to the Earth’s snowball state. As this takes place, the smaller *γ*, the smaller noise intensity *ε* that determines this breakdown transition (the deterministic attractor is a limit cycle for figure 4*a* whereas it is a stable equilibrium for figure 4*b*).

**Figure 5.**
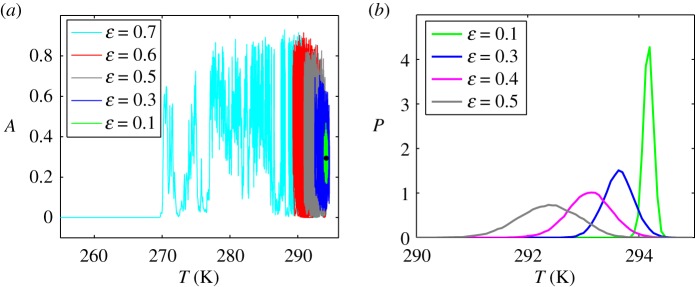
Noise-induced shift of the global temperature and probability density function for *γ*=0.35.

Some details of this transformation of the stochastic dynamics of system (4.1) with increasing noise are shown in figure 5 for *γ*=0.35. Here, the stochastic phase trajectories and probability density functions for different noise intensities *ε* are shown. In the case of small *ε*, the stochastic system is localized in the temperature range of approximately 290≲T≲300 K. As this takes place, a noise-induced shift to lower global temperatures arises. The noise induces therewith substantial fluctuations in the vegetation land cover *A*. The noise-induced shift occurring at large intensities (*ε*=0.7 in figure 5) leads to random walks, which ultimately end up in the catastrophic state of the snowball Earth. The probability density function *P* becomes more broad and flat with decreasing mean value of global temperature *T* (figure 5*b*). This confirms the effect of a noise-induced shift of the global temperature shown in figure 5*a*. What is more, in the case of moderate noises, this statistically confirms the vegetation–climate system fluctuations within a sufficiently narrow temperature interval and at the same time within a broad interval of vegetation cover changes.

## 5. Conclusion

In summary, we consider a simple climate–vegetation model consisting of a land surface covered by some vegetation and ocean. Temperature changes induce the growth of vegetation and alter the Earth’s surface albedo, which in turn modifies the energy balance and, thus, induces temperature changes. At first, the nonlinear feedback model coupling the Earth’s climate and vegetation is studied in the idealized case of deterministic dynamics. This analysis details the main features of deterministic dynamics and, in particular, it demonstrates the system attractors. Further we show that the evolutionary behaviour of the climate–vegetation system drastically depends on externally induced stochastic forcing which, in turn, can be caused by different anthropogenic and natural processes influencing the nonlinear dynamics.

Let us summarize below the main features of the initial deterministic climate–vegetation model [34]. This model possesses two stable states which are divided by a separatrix going across the unstable equilibrium. These stable states are either the limit cycle and equilibrium or two equilibria depending upon the value of the vegetation death rate. The system attractor of snowball Earth therewith is always the stable equilibrium. Another stable state describing the warm climate is the limit cycle embracing the unstable equilibrium or the stable equilibrium appearing as a result of their merging. An important point is that the oscillation periods of the global average temperature and the land fraction covered by vegetation decrease with increasing vegetation death rate.

Note that deterministic phase trajectories going to two opposite climate–vegetation states can be located very close to each other. As a result, even small external stochastic forcing can throw the system across the separatrix, and two opposite stable climate–vegetation states (having the warm and cold climate) become stochastically interconnected.

What is more, this transition between system attractors becomes steeper when the vegetation death rate increases. In addition, increasing this rate gives the greater noise intensities leading to the snowball state.

An important outcome of our stochastic analysis lies in the fact that the climate–vegetation system undergoes a noise-induced shift into the range of smaller global average temperatures where it fluctuates with a broad range of possible land fractions covered by vegetation. This shift can be responsible for substantial climate changes. Therefore, the coupled climate–vegetation model should be taken into account in conjunction with more precise and complete models of climate variability incorporating diverse interactions between the ocean, ice and atmosphere [24–26,37–40].

## Data accessibility

This article has no additional data.
